# Isomer‐Specific Vibrational Spectroscopy of Microhydrated Lithium Dichloride Anions: Spectral Fingerprint of Solvent‐Shared Ion Pairs

**DOI:** 10.1002/cphc.202100170

**Published:** 2021-05-04

**Authors:** Arghya Chakraborty, Sonja Schmahl, Knut R. Asmis

**Affiliations:** ^1^ Wilhelm-Ostwald-Institut für Physikalische und Theoretische Chemie Universität Leipzig Linnéstrasse 2 D-04103 Leipzig Germany

**Keywords:** anharmonicity, hydrogen bond, infrared photodissociation, microhydration, salt dissolution

## Abstract

The vibrational spectroscopy of lithium dichloride anions microhydrated with one to three water molecules, [LiCl_2_(H_2_O)_1–3_]^−^, is studied in the OH stretching region (3800–2800 cm^−1^) using isomer‐specific IR/IR double‐resonance population labelling experiments. The spectroscopic fingerprints of individual isomers can only be unambiguously assigned after anharmonic effects are considered, but then yield molecular level insight into the onset of salt dissolution in these gas phase model systems. Based on the extent of the observed frequency shifts Δν^OH^ of the hydrogen‐bonded OH stretching oscillators solvent‐shared ion pair motifs (<3200 cm^−1^) can be distinguished from intact‐core structures (>3200 cm^−1^). The characteristic fingerprint of a water molecule trapped directly in‐between two ions of opposite charge provides an alternative route to evaluate the extent of ion pairing in aqueous electrolyte solutions.

## Graphical abstract

**Spectroscopic identification of solvent‐shared ion pairs**: Isomer‐specific vibrational spectra of microhydrated lithium dichloride anions in the gas phase allow to unambiguously distinguish between contact and solvent‐shared ion pair cores. Red‐shifted OH stretching bands are corelated to characteristic ion‐water and water‐water interactions based on a comparison to anharmonic IR spectra from electronic structure calculations.

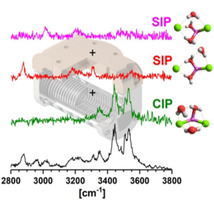

The interactions governing ion hydration are of fundamental interest as they have direct implications to diverse fields ranging from biochemistry^[1]^ to chemical engineering^[2]^ to atmospheric chemistry.^[3]^ Significant progress has been made in recent years regarding the experimental characterization of the specificity and the spatial extend of ion‐water interactions.[^4^, ^5^, ^6^] However, obtaining molecular‐level information on salt dissolution is obscured by the heterogeneity as well as the highly dynamic nature of the hydrogen‐bonded networks comprising aqueous solutions.^[7]^ One of the central challenges, currently, is assessing the extent of ion pairing in aqueous electrolyte solutions, typically discussed in the context of contact (CIP), solvent‐shared (SIP) and solvent‐separated ion pair (2SIP) formation.[^5^, ^8^, ^9^]

Such motifs are rather elusive in solution, but can, in principle, be isolated and characterized in the form of microhydrated gas phase clusters.^[10]^ In this context, anion photoelectron spectroscopy in combination with high level electronic structure calculations has proven particularly useful for obtaining a molecular level insight into the first stages of salt dissolution in such model systems.^[11]^ However, correlating measured detachment energies with a particular ion pair motif, specifically, when multiple isomers are present, is challenging. More detailed structural information can be extracted using infrared photodissociation (IRPD) spectroscopy, in particular, when performed isomer‐specifically.^[12]^ So far, IRPD studies on microhydrated cluster ions have mainly focused on the characterization of CIPs.^[13]^ Here, we use cryogenic ion trap vibrational spectroscopy,^[14]^ a particularly powerful variant of IRPD spectroscopy, to study the onset of salt dissolution in microhydrated lithium dichloride anions (LiCl_2_)^−^ and to identify the spectroscopic fingerprint of SIPs for the first time.

IRPD spectra of D_2_‐tagged [LiCl_2_(H_2_O)_*n*_]^−^ anions (*n*=1–3) measured in the OH stretching region (3800‐2800 cm^−1^) are shown in Figure 1 and peak positions are listed in Table S1 provided in the Supporting Information (SI). The three spectra are surprisingly complex, considering the limited number of OH oscillators in each system, namely two (*n=*1), four (*n*=2) and six (*n*=3). A comparison to previously published IRPD spectra of Ar‐tagged Cl^−^(H_2_O)_1–3_ and Li^+^(H_2_O)_1–3_ reveals no obvious similarities, but does indicate that the water‐chloride interaction changes substantially and non‐monotonically upon sequential addition of up to three water molecules to (LiCl_2_)^−^.[^15^, ^16^] Moreover, the previous data is helpful in defining the three characteristic absorption regions indicated in Figure 1. Excitation of free OH oscillators is found in region I (>3650 cm^−1^). All three systems show absorptions in this region, of which band *b_1_* (*n*=2) is considerably more intense than the corresponding features *a_1_* and *c_1_*. Region II (3650–3200 cm^−1^) covers OH oscillators involved in water‐water as well as weaker chloride‐water hydrogen bonds. The *n*=1 and *n*=3 spectra display strong absorptions in this region. In contrast, the *n*=2 spectrum shows its most intense bands in region III (<3200 cm^−1^), which is characteristic for strong chloride‐water interactions. Summarizing, the spectral signature of the microhydrated [LiCl_2_(H_2_O)_1–3_]^−^ anions in the OH stretching region extends over a spectral range of up to ∼900 cm^−1^, indicating the presence of multiple water binding motifs. Indeed, isomer‐specific IR^2^MS^2^ measurements (*vide infra*) reveal that multiple isomers contribute to each of the three spectra shown in Figure 1.

**Figure 1:**
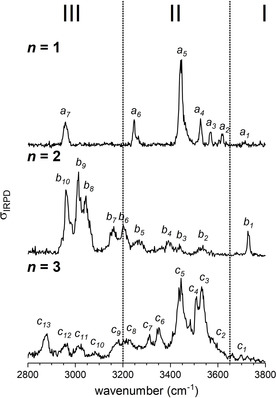
IRPD spectra of D_2_‐tagged [LiCl_2_(H_2_O)_*n*_]^−^ (*n*=1–3) measured at an ion trap temperature of 13 K in the spectral region from 2800 to 3800 cm^−1^. Dashed lines separate the free (I) and the hydrogen‐bonded OH stretching regions (II, III), of which the latter can be divided further into the intact (II) and the solvent‐shared ion‐core regions (III). See Table S1 for band positions and assignments.

In order to identify these contributing isomers, we performed electronic structure calculations. We initially determined minimum‐energy structures and harmonic IR spectra using density functional theory (DFT) calculations (see methodology, Table S2 and Figure S3–S6 in the SI). However, the agreement of the harmonic frequencies with the experimental ones was very poor. Inclusion of anharmonic effects is necessary and we did this using second‐order perturbation theory (VPT2), which improved the agreement considerably (see Figures S3 to S6 in the SI). The best agreement was found for VPT2/MP2 anharmonic spectra and therefore the corresponding minimum‐energy structures of the relevant low energy isomers are shown in Figures 2–4. A cosine similarity score analysis consolidating the structural assignment is provided in Table S4. Additional higher‐energy structures can be found in Figure S2 of the SI.

**Figure 2:**
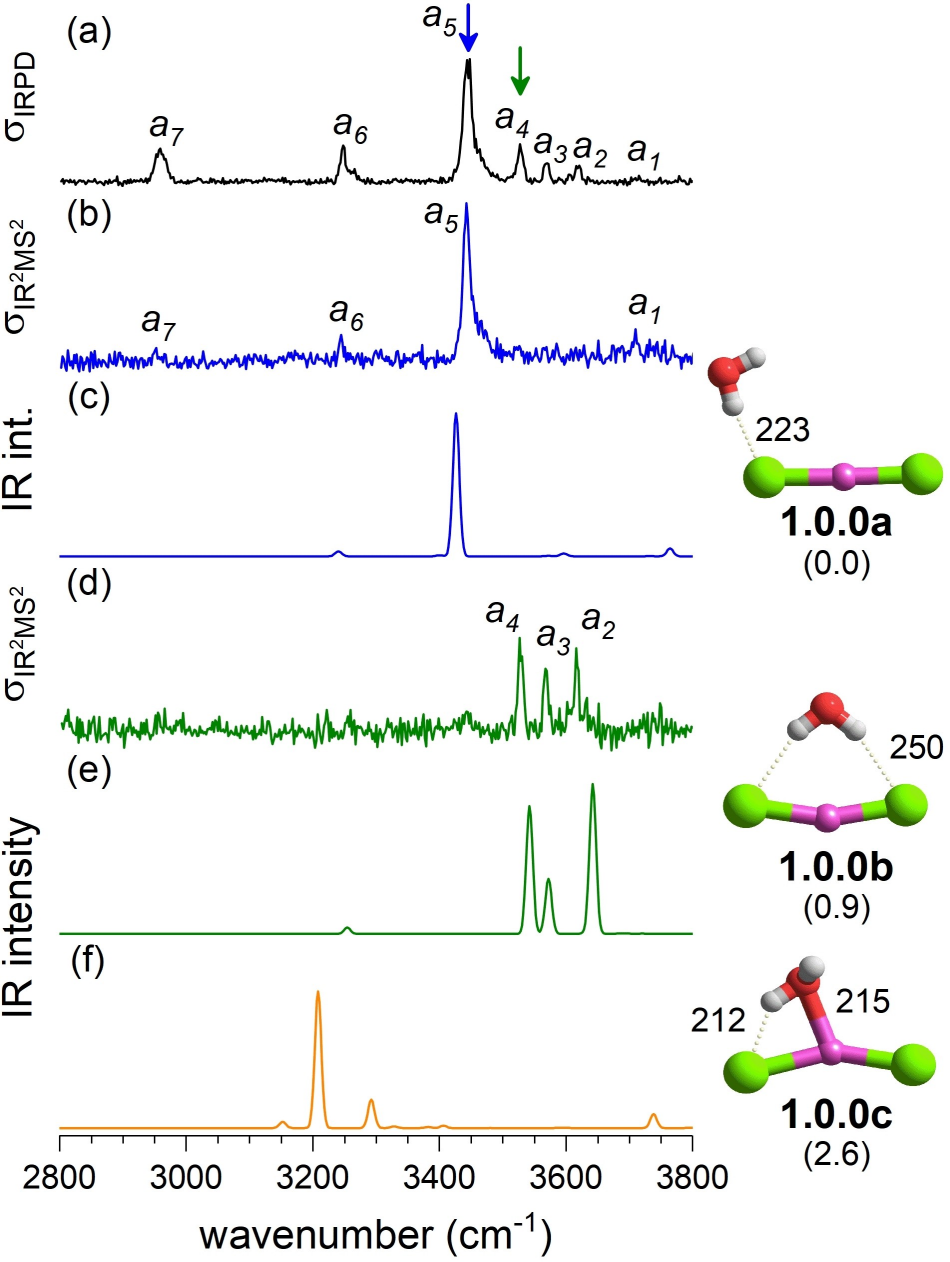
Comparison of the IRPD spectrum (trace a) of D_2_‐tagged [LiCl_2_(H_2_O)]^−^ to IR^2^MS^2^ spectra obtained by probing at band *a_5_* (b) and *a_4_* (d), as indicated by color coded arrows. Anharmonic VPT2/MP2/6‐311++G (2df,2pd) spectra of the three lowest energy isomers **1.0.0 a**, **1.0.0 b** and **1.0.0 c** are presented in trace c, e and f, respectively. Minimum‐energy structures, ZPE‐corrected relative energies Δ*E*_0,anh_ (in kJ/mol) and characteristic bond lengths (in pm) are also shown. See Table S1 for band positions and assignments.

**Figure 3:**
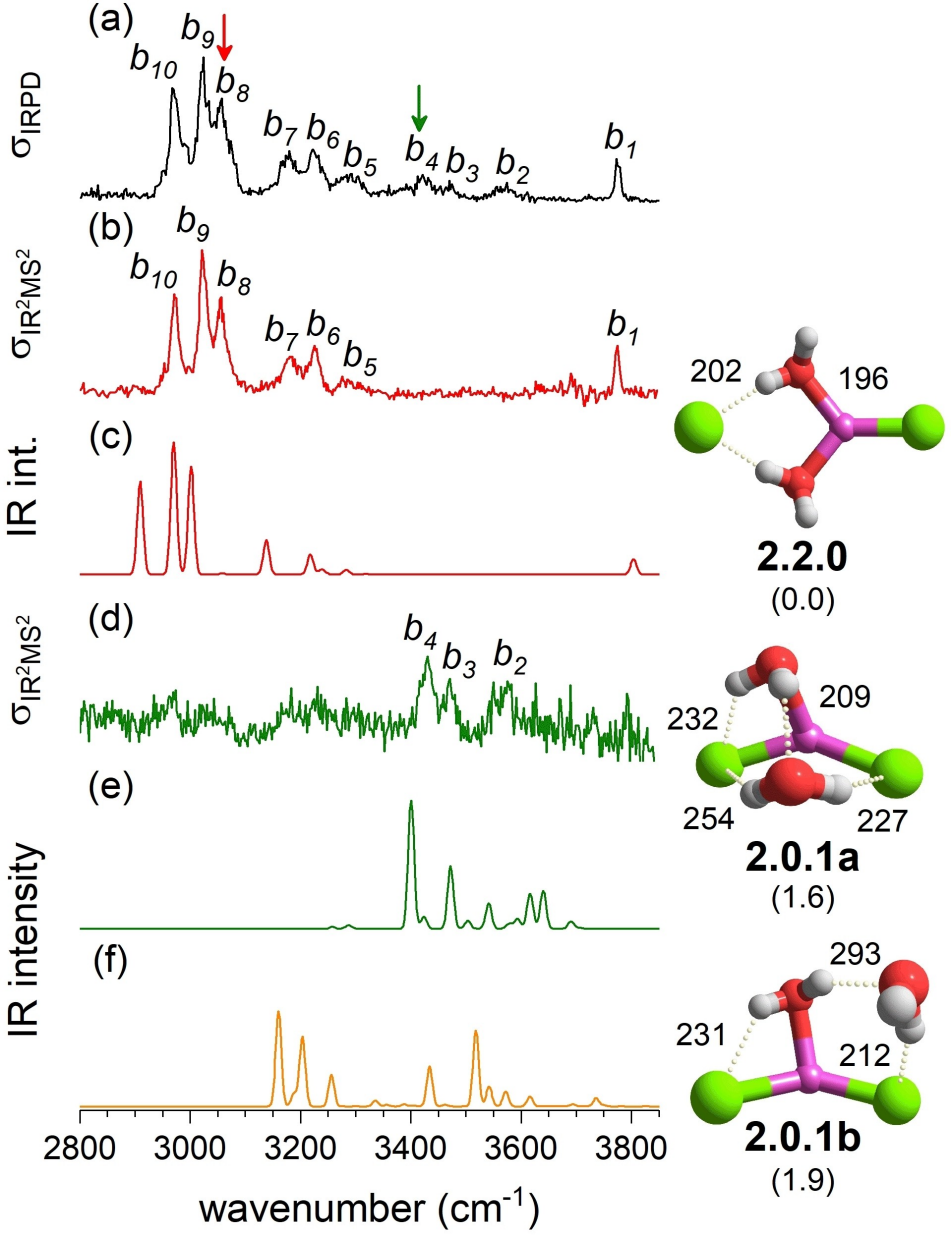
Comparison of the IRPD spectrum (trace a) of D_2_‐tagged [LiCl_2_(H_2_O)_2_]^−^ to IR^2^MS^2^ spectra obtained by probing at band *b_8_* (b) and *b_4_* (d), as indicated by color coded arrows. Anharmonic VPT2/MP2/6‐311++G (2df,2pd) spectra of the three most stable isomers **2.2.0**, **2.0.1 a** and **2.0.1 b** are presented in trace c, e and f, respectively. Minimum‐energy structures, ZPE‐corrected relative energies Δ*E*_0,anh_ (in kJ/mol) and characteristic bond lengths (in pm) are also shown. See Table S1 for band positions and assignments.

**Figure 4:**
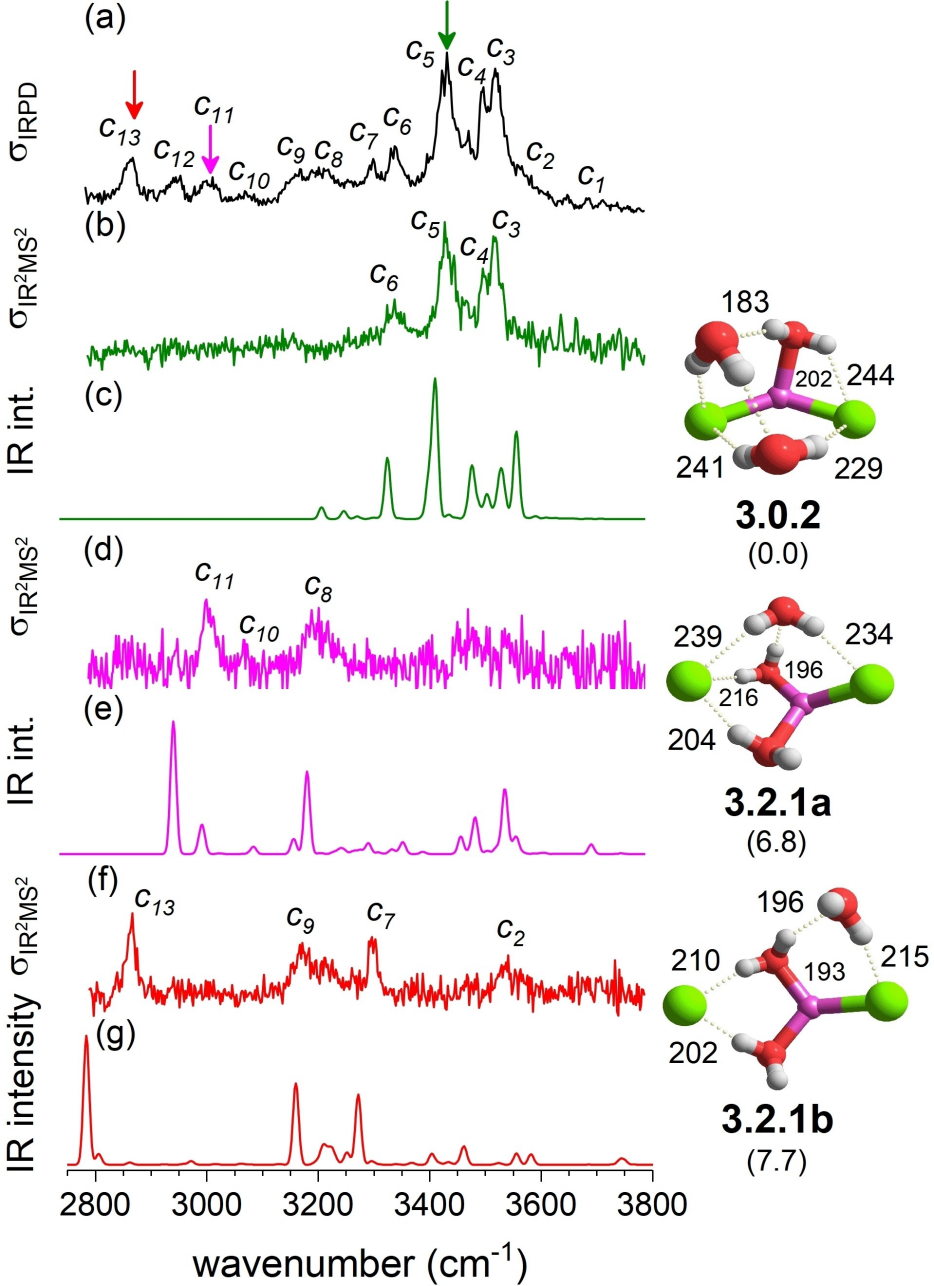
Comparison of the IRPD spectrum (trace a) of D_2_‐tagged [LiCl_2_(H_2_O)_3_]^−^ to IR^2^MS^2^ spectra obtained by probing at band *c_5_* (b), *c_11_* (d) and *c_13_* (f), as indicated by color‐coded arrows. Anharmonic VPT2/MP2/6‐311++G(2df,2pd) spectra of the three most stable isomers **3.0.2**, **3.2.1 a** and **3.2.1 b** are presented in trace c, e and g, respectively. Minimum‐energy structures, ZPE‐corrected relative energies Δ*E*_0,anh_ (in kJ/mol) and characteristic bond lengths (in pm) are also shown. See Table S1 for band positions and assignments.

Relative zero‐point energy (ZPE) corrected MP2 energies Δ*E*_0,anh_ and selected geometric parameters are summarized in Table 1. Isomers are labelled using the ***m.bw.i*** nomenclature scheme, where *m* refers to the number of water molecules, *bw* to the number of inter‐ion bridging water molecules and *i* to the number of water‐water hydrogen bonds. In addition, the letters *a‐z* are used in ascending order to differentiate between multiple (***m.bw.i***) isomers according to Δ*E*_0,anh_.

**Table 1:** MP2/6‐311++G(2df,2pd) relative energies Δ*E*, ZPE‐corrected relative energies Δ*E*_0,anh_ (both in kJ/mol), vertical detachment energies VDE (in eV), interion distances *r*_Li−Cl_ (in pm) and ion‐core angle α_Cl−Li−Cl'_ (in °) of low energy isomers for [LiCl_2_(H_2_O)_*n*_]^−^ with *n*=0–3.

Label	Δ*E*	Δ*E*_0,anh_^[a]^	*r*_Li−Cl_/*r*_Li−Cl′_^[b]^	α_Cl−Li−Cl'_	VDE
0.0.0			216/216	180	
1.0.0a	2.3	**0.0**	218/215	178	6.21
1.0.0b	**0.0**	0.9	216/216	161	6.33
1.0.0c	1.6	2.6	229/218	154	6.19
1.1.0	32.6	28.1	420/213	164	5.54
					
2.2.0	1.3	**0.0**	380/218	155	6.08
2.0.1a	**0.0**	1.6	227/221	138	6.13
2.0.1b	1.5	1.9	225/223	147	6.09
2.0.0	6.3	7.1	231/231	129	6.02
2.0.1c	20.5	16.2	219/214	177	6.39
					
3.0.2	**0.0**	**0.0**	226/223	136	6.45
3.2.1a	7.9	6.8	344/220	142	6.26
3.2.1b	11.8	7.7	366/222	118	5.95
3.2.1c	14.0	8.9	371/222	151	5.90
3.3.0	16.6	11.0	366/220	180	–

[a] ZPE‐corrected energies derived from anharmonic MP2/VPT2 frequencies. [b] Interion distances of solvent‐shared motifs underlined.

The lithium dichloride anion, (ClLiCl)^−^, exhibits a linear *D*_∞h_ geometry.^[17]^ Addition of the first water molecule leads to three isomers that lie within less than 3 kJ/mol of each other. All three exhibit an intact but bent ion core. The global ground state structure **1.0.0 b** (see Figure 2) possesses a double donor (DD) water molecule (DD‐H_2_O), which forms a hydrogen bond with each of the two chloride anions. It is followed by the single donor (D) structures **1.0.0 c** (+1.6 kJ/mol) and **1.0.0 a** (+2.3 kJ/mol), which differ in the strength of D‐H_2_O⋅⋅⋅Li^+^ interaction. Solvent‐shared structures, like **1.1.0** (Table 1), are predicted over 25 kJ/mol higher in energy. The consideration of ZPE changes the relative stability of the intact‐core isomers and leads to the ordering indicated by letter index, i. e. **1.0.0 a** is more stable than **1.0.0 b** (+0.9 kJ/mol) and **1.0.0 c** (+2.6 kJ/mol).

IR^2^MS^2^ spectra of D_2_‐tagged [LiCl_2_(H_2_O)]^−^ probed at the wavelengths corresponding to peaks *a_5_* (3444 cm^−1^) and *a_4_* (3529 cm^−1^) are shown in Figure 2b and 2d, respectively. They show that two isomers contribute to the IRPD spectrum of *n*=1 (see Figure 2a). Comparison of the experimental spectra to the anharmonic spectra (Figure 2c and 2e) unambiguously reveals **1.0.0 a** as the dominant isomer and **1.0.0 b** also contributes, in agreement with the predicted energy ordering. Trace 2b is characterized by bands *a_1_*, *a_5_*, *a_6_* and *a_7_*, which are assigned to excitation of the fundamentals of the free (3709 cm^−1^) and the hydrogen‐bonded OH stretching mode (3444 cm^−1^), the H_2_O bending overtone (3246 cm^−1^) as well as the stretching mode of the D_2_ tag (2958 cm^−1^), respectively (Table S1). The spectrum of the less abundant isomer exhibits a triplet of peaks (*a_2_*‐*a_4_*), which are characteristic for the DD‐H_2_O motif. We assign *a_2_* and *a_4_* to the excitation of the antisymmetric and symmetric H_2_O stretching modes, respectively, and *a_3_* to the combination band involving the symmetric H_2_O stretching and lowest energy libration mode. Contribution of additional low energy isomers, like **1.0.0 c** (see Figure 2f), to the IRPD spectrum (Figure 2a) is not evident.

Addition of a second water molecule (see Figure 3) is sufficient to bring solvent‐shared structures, like **2.2.0** (+1.3 kJ/mol), close in energy to the intact‐core structures **2.0.1 a** (global minimum) and **2.0.1 b** (+1.5 kJ/mol). Indeed, when ZPE is considered, **2.2.0** is predicted slightly more stable than **2.0.1 a** (1.6 kJ/mol) and **2.0.1 b** (1.9 kJ/mol). **2.2.0** contains two characteristic bridging D‐H_2_O′s, each donating a single strong hydrogen bond (*r*_H⋅⋅⋅Cl_=202 pm) to the same chloride anion. This results in a bent (α=155°) *C*_s_ solvent‐shared structure with interion distances *r*_Li‐Cl_ of 380 pm and 218 pm, i. e. +76 % and +1 % with respect to **1.0.0 a**. The two intact‐core structures correspond to different combinations of the corresponding *n*=1 motifs, of which **2.0.1 a** is more compact, due to an additional, very weak ion‐water hydrogen bond (*r*_H⋅⋅⋅Cl_=254 pm) donated by the acceptor double‐donor water molecule (ADD‐H_2_O) accepting the hydrogen bond from the DD‐H_2_O. Structures, in which the water molecules predominantly interact with one of the chloride anions (**2.0.1 c**), like in Cl^−^(H_2_O)_2_, are found at least 15 kJ/mol higher in energy. Note, addition of water, in general, decreases the interion angle and this trend is continued for *n*=3.

IR^2^MS^2^ spectra of D_2_‐tagged [LiCl_2_(H_2_O)_2_]^−^ probed at the wavelengths corresponding to peaks *b_8_* (3045 cm^−1^) and *b_4_* (3394 cm^−1^) are shown in Figure 3b and 3d, respectively. They show that two isomers also contribute to the IRPD spectrum of *n*=2 (Figure 3a). However, the nature of the spectrum of the dominant *n*=2 isomer is significantly different from all previously discussed spectra. Beside the surprisingly intense free OH stretching band at 3729 cm^−1^ (*b_1_*) and substantial IR activity below 3200 cm^−1^, it shows little to no IR activity in region II (3650–3200 cm^−1^). Comparison to the simulated IR spectra in Figure 3 leaves no doubt that this is the characteristic IR signature of the solvent‐shared structure **2.2.0**. This identifies bands *b_9_* (3014 cm^−1^ and *b_10_* (2960 cm^−1^) ) as the in‐phase and out‐of‐phase stretching modes of the two hydrogen‐bonded OH oscillators of the two bridging D‐H_2_O′s, predicted at 2962 cm^−1^ and 2904 cm^−1^, respectively (see Table S1). The peaks *b_6_*–*b_8_* are combination bands involving these two fundamentals and low‐frequency librations. *b_5_* is assigned to the D‐H_2_O bend overtones. The weaker signals above 3350 cm^−1^ (see Figure 3d) are attributed to a second isomer. This is likely the intact‐core structure **2.0.1 a** (Figure 3d), which is predicted next in energy and exhibits three IR‐active hydrogen‐bonded OH stretching fundamentals at 3506 cm^−1^, 3440 cm^−1^ and 3372 cm^−1^ (see Table S1), i. e. in close vicinity to the experimentally observed bands at 3536 cm^−1^ (*b_2_*), 3437 cm^−1^ (*b_3_*) and 3397 cm^−1^ (*b_4_*).

Interestingly, addition of the third water molecule stabilizes the intact‐core structure **3.0.2** (see Figure 4) substantially relative to solvent‐shared structures **3.2.1 a**–**c** (>8 kJ/mol). Structure **3.0.2**, which represents the global minimum, independent of ZPE considerations, can be seen as a combination of the motifs found in **2.0.1 b** and **1.0.0 b** and contains the shortest water‐water hydrogen bond (*r*_H⋅⋅⋅O_=183 pm) of all structures considered here, while the ion‐water hydrogen bonds are among the longest and hence weakest. Even though the ion core remains intact, the interion distances *r*_Li‐Cl_ have increased slightly, i. e. by 3 % and 5 % (with respect in **0.0.0**) and the interion angle has decreased to 136°. The solvent‐shared structures **3.2.1 a**, **3.2.1 b** and **3.2.1 c** are characterized by short and strong ion‐water hydrogen bonds alike **2.2.0**. The isomer **3.3.0**, which contains three Li⋅⋅⋅(OH_2_)⋅⋅⋅Cl solvent‐bridges, is found at least 11 kJ/mol higher in energy.

IR^2^MS^2^ spectra of D_2_‐tagged [LiCl_2_(H_2_O)_3_]^−^ probed at the wavenumbers corresponding to peaks *c_5_* (3443 cm^−1^), *c_11_* (3016 cm^−1^) and *c_13_* (2878 cm^−1^) are shown in Figure 4b, 4d and 4 f, respectively. They show that three isomers contribute to the IRPD spectrum of *n*=3 (see Figure 4a). The most abundant isomer (Figure 4b) is associated with an intact‐core structure, while the other two (Figure 4d and 4 f) must be solvent‐shared structures, as only they exhibit characteristic signals below 3200 cm^−1^. Simulated IR spectra of the three lowest energy isomers **3.0.2**, **3.2.1 a** and **3.2.1 b** (Figure 4c, 4e and 4 g) do indeed display the best agreement with the corresponding experimental spectra. Bands *c_3_* to *c_6_* are associated with the intact‐core structure **3.0.2** and are assigned to excitation of the antisymmetric and symmetric H_2_O stretching modes, respectively, of the DD‐H_2_O. This water molecule is involved in the strongest water‐water hydrogen bond (183 pm) of all systems discussed here. Bands *c_4_* and *c_5_* are attributed to the in‐phase antisymmetric and symmetric stretching modes of the two ADD‐H_2_O, of which one donates a hydrogen bond to each chloride anion (see Table S1). The markedly red‐shifted bands *c_11_* and *c_13_* are the signature OH stretching bands of the D‐H_2_O′s located between one of the two Li^+^/Cl^−^ ion pairs as part of the solvent‐shared motifs **3.2.1 a** and **3.2.1 b**. Of these bands, *c_13_*, observed at 2878 cm^−1^ (predicted at 2784 cm^−1^), is the most red‐shifted and hence associated with the shortest/strongest anion‐water hydrogen bond (198 pm) in **3.2.1 b**. Bands *c_11_* and *c_10_* are attributed to **3.2.1 a** and the latter is a combination band involving a low‐frequency mode.

Based on the satisfactory assignment of all the major spectral features we can now derive some more general insights. The microhydration motifs observed in these relatively small clusters are the result of a delicate balance of ion‐ion, ion‐water and water‐water interactions. All three interactions are competitive which is exemplified by two observations: (1) Only two water molecules are needed to stabilize the SIP motif, i. e. strong ion‐water interactions can more than compensate a significant decrease in attractive ion‐ion interaction. However, this SIP motif is rather rigid. (2) Therefore, addition of a third water molecules leads to re‐stabilization of the CIP motif, because this allows for the formation of a more extended hydrogen‐bonded network involving two water‐water interactions and avoiding free OH groups.

The vibrational spectra of microhydrated salt ions appear remarkably complex for two reasons: the presence of multiple isomers and anharmonicity. That multiple competing structures are present which may not come as such a surprise, considering multiple competing water binding motifs. It is noteworthy that, similar to recent studies on microhydrated sulfate dianions,^[18]^ we find that ZPE corrections are important for reliably predicting the lowest energy isomer. The VPT2/MP2 method yields ZPE‐corrected energies reflecting the observed isomer abundances, i. e. the most abundant isomer observed is also the thermodynamically most stable one. Moreover, anharmonic effects are remarkably well reproduced with respect to the observed frequency shifts and relative IR intensities. This also suggests that the isomerization barriers are still high with respect to the average internal energy. At higher temperatures dynamic effects need to be considered.^[19]^

The present results are also in agreement with the previously identified correlation^[20]^ regarding red‐shifts in the stretching frequency of the hydrogen‐bonded OH oscillators with respect to the averaged OH stretching frequency of a free water molecule. Experimentally determined red‐shifts Δν_OH_ are plotted against the corresponding, theoretically obtained, change in the vibrationally averaged bond length Δ*R*_0_ (with respect to *R*_0_ of an isolated water molecule) in Figure S9. The ratio Δν/Δ*R*_0_=−18.6 cm^−1^/10 pm found in our current study is similar to the computed values for pure water clusters (−20.2 cm^−1^/10 pm)^[21]^ and water clusters with incorporated halide and hydronium ions (−19.1 cm^−1^/10 pm).^[20]^

Isomer‐specific vibrational spectra of microhydrated salt complexes allow to differentiate between SIP and CIP motifs based on the presence (or absence) of characteristically red‐shifted OH stretching frequencies of shared water molecules below 3200 cm^−1^. The characteristic fingerprint of a water molecule trapped directly in‐between two ions of opposite charge falls into a spectral region in which the absorption of pure water drops off markedly^[15]^ and therefore may provide an alternative route to evaluate the extent of SIP formation in aqueous electrolyte solutions. In contrast, vertical detachment energies (Table 1) are not so distinctive and therefore it will be difficult to distinguish these motifs using anion photoelectron spectroscopy.

We also evaluated, in how far the spectral signatures in the far‐IR spectral region would be useful in identifying CIPs in the gas phase (see Figure S10 of SI). This is the spectral region of the ion rattling motion in solution, which makes it possible to quantify the extent of ion pairing in highly concentrated electrolytes.^[6]^ For protons a corresponding gas phase feature has been identified in the spectra of larger protonated water clusters.^[22]^ The degree of hydration in the present systems is too small to form hydration cages, but IR‐active LiCl interion vibrations (Table S3) are also predicted in this spectral range (<700 cm^−1^). However, these bands are not diagnostic for a particular ion pair motif. Moreover, this spectral range quickly suffers from spectral congestion due to water librations as the number of water molecules increases.

In conclusion, experiments on microhydrated salt clusters will prove helpful in testing, how well computational models deal with reproducing solvent polarization effects. Typically, simulations on larger biomolecular systems in aqueous electrolyte solutions are performed using non‐polarizable force fields, due to computational constraints.^[23]^ However, such models are not able to even qualitatively reproduce the aqueous solubility of salts containing high charge density ions (e. g. Li^+^).^[24]^ In principle, such problems can be addressed using charge scaling schemes, which however, introduce other complications.^[9]^ Testing these schemes on gas phase clusters, like the ones studied here, which are amenable to high level computations, should make it possible to evaluate their applicability.

## Conflict of interest

The authors declare no conflict of interest.

## Supporting information

As a service to our authors and readers, this journal provides supporting information supplied by the authors. Such materials are peer reviewed and may be re‐organized for online delivery, but are not copy‐edited or typeset. Technical support issues arising from supporting information (other than missing files) should be addressed to the authors.

Supplementary
