# Supplementary material for: Isomer‐Specific Vibrational Spectroscopy of Microhydrated Lithium Dichloride Anions: Spectral Fingerprint of Solvent‐Shared Ion Pairs
Source: Chemphyschem. 2021 May 4;22(11):1036–41. doi: 10.1002/cphc.202100170 (PMC8252531; doi:10.1002/cphc.202100170)
Supplement: Supplementary file 1 — Supplementary [file CPHC-22-1036-s001.pdf]

ChemPhysChem

Supporting Information

Isomer-Specific Vibrational Spectroscopy of Microhydrated Lithium Dichloride Anions: Spectral Fingerprint of Solvent-Shared Ion Pairs

Arghya Chakraborty, Sonja Schmahl, and Knut R. Asmis*

Table of Contents

	Page
**Methodology.** Experimental and computational	3-4
**Figure S1.** Quadrupole mass spectrum	6
**Figure S2.** Minimum-energy structures of $[LiCl_2(H_2O)_n]^-$ (*n* = 1-3) Isomers	7
**Figure S3.** $[LiCl_2(H_2O)]^-$: Experimental (IRPD and IR^2MS^2) spectra *vs.* DFT harmonic and MP2 and DFT anharmonic IR spectra of assigned isomers	8
**Figure S4.** $[LiCl_2(H_2O)_2]^-$: Experimental (IRPD and IR^2MS^2) spectra *vs.* DFT harmonic and MP2 and DFT anharmonic IR spectra of assigned isomers	9
**Figure S5.** $[LiCl_2(H_2O)_3]^-$: Experimental (IRPD and IR^2MS^2) spectra *vs.* DFT harmonic and MP2 and DFT anharmonic IR spectra of isomer **3.0.2**	10
**Figure S6.** $[LiCl_2(H_2O)_3]^-$: Experimental (IRPD and IR^2MS^2) spectra *vs.* DFT harmonic and MP2 and DFT anharmonic IR spectra of **3.2.1 a** and **3.2.1b**.	11
**Figure S7.** VPT2/MP2 anharmonic IR spectra of $[LiCl_2(H_2O)_2]^-$ isomers	12
**Figure S8.** VPT2/MP2 anharmonic IR spectra of $[LiCl_2(H_2O)_3]^-$ isomers	13
**Figure S9**. Structure - IR frequency correlation diagram	14
**Figure S10.** MP2 harmonic spectra in the 0-700 cm^{-1} region	15
**Table S1.** IRPD and IR^2MS^2 band positions, anharmonic vibrational frequencies and band assignments .	16
**Table S2.** DFT and MP2 computed relative energies ΔE and ZPE-corrected relative energies ΔE_0 of low-energy isomers	18
**Table S3.** MP2 harmonic IR frequencies of Cl-Li-Cl core vibrations	19
**Table S4.** Cosine similarity score analysis	20
**Table S5**. Cartesian coordinates of MP2 minimum-energy structures	22-27
**References**	4-5

Methodology:

Experimental Methods. IRPD experiments were performed on the Leipzig 6 K ring-electrode trap triple mass spectrometer described previously.[1] Microhydrated lithium dichloride anions were generated by a nanospray ion source from 10 mM lithium chloride solutions in 1:1 water/acetonitrile mixture (see Figure S1). The beam of anions is skimmed, thermalized to room temperature in a helium-filled radio-frequency (RF) ion-guide, and then mass-selected using a quadrupole mass filter. The mass-selected $[LiCl_2(H_2O)_n]^-$ ions are trapped in a RF ring-electrode ion trap held at a 13 K, where they are thermalized to the ambient temperature of the trap and messenger tagged with D_2 (He used for bare ions).

IRPD spectra are measured using the IR^1MS^2 technique, which allows for a background-free photofragment detection.[2] Ions with a particular m/z value are irradiated by a properly timed, widely wavelength tunable (750-7000 cm^{-1}) IR laser pulse (bandwidth: ~ 2.5 cm^{-1}), supplied by an optical parametric oscillator/amplifier (LaserVision: OPO/OPA/$AgGaSe_2$) laser system.[3] IRPD spectra are measured by continuously scanning the laser wavelength monitored online using a HighFinesse WS6-600 wavelength meter with a scan speed such that an averaged TOF mass spectrum (over 100 laser shots) is obtained every 2 cm^{-1}. Typically, at least such five scans are measured and averaged and the photodissociation cross section σ_{IRPD} is determined as described previously.[1] Double-resonance IR^2MS^2 spectra using the ion-dip technique are obtained by employing two tunable IR lasers in a pump–probe approach and tandem mass selection stages.[4] Briefly, the method is sensitive to laser-induced population changes of isomers that are maintained longer than the delay between pump and probe laser. The irradiation of the ions by two laser pulses creating two sets of photofragment ions and their detections have been previously explained.[4]

Computational Methods. Electronic structure calculations were performed using the Gaussian 16 rev. A.03 programme.[5] MP2/6-311++G(2df,2pd) minimum-energy geometries were determined and minima were validated by a subsequent harmonic frequency analysis. For specific structures we performed anharmonic frequency calculations using the second-order vibrational perturbation theory (VPT2) method as implemented by Bloino and Barone.[6] Spectra are determined from the computed stick spectra, which are convoluted with a Gaussian line shape function (FWHM =12 cm^{-1}) for better comparability. Zero-point energy (ZPE) corrected relative energies $\Delta E_{0,\mathrm{anh}}$ were determined by adding the anharmonic VPT2/MP2 ZPE to the MP2 energy.

Reference

[1] a) N. Heine, K. R. Asmis, *Int. Rev. Phys. Chem.* **2015**, *34*, 1; b) K. R. Asmis, *Int. Rev. Phys. Chem.*, **2016**, *35*, 507.

[2] M. Pahl, M. Mayer, M. Schneider, D. Belder, K. R. Asmis, *Anal. Chem.* **2019**, *91*, 3199.

[3] D. J. Goebbert, T. Wende, R. Bergmann, G. Meijer, K. R. Asmis, *J. Phys. Chem. A* **2009**, *113*, 5874.

[4] N. Heine, M. R. Fagiani, M. Rossi, T. Wende, G. Berden, V. Blum, K. R. Asmis, *J. Am. Chem. Soc.* **2013**, *135*, 8266.

[5] M. J. Frisch, G. W. Trucks, H. B. Schlegel, G. E. Scuseria,M. A. Robb, J. R. Cheeseman, G. Scalmani, V. Barone,G. A. Petersson and H. Nakatsuji **, 2016.**

[6] J. Bloino, V. Barone, *J. Chem. Phys.* **2012**, *136*, 124108.

[7] L. J. M. Kempkes, J. Martens, G. Berden, K. J. Houthuijs, J. Oomens, *Faraday Discuss.* **2019**, *217*, 434–452.

[8] Gaussian 16, Revision C.01, M. J. Frisch, G. W. Trucks, H. B. Schlegel, G. E. Scuseria, M. A. Robb, J. R. Cheeseman, G. Scalmani, V. Barone, G. A. Petersson, H. Nakatsuji, X. Li, M. Caricato, A. V. Marenich, J. Bloino, B. G. Janesko, R. Gomperts, B. Mennucci, H. P. Hratchian, J. V. Ortiz, A. F. Izmaylov, J. L. Sonnenberg, D. Williams-Young, F. Ding, F. Lipparini, F. Egidi, J. Goings, B. Peng, A. Petrone, T. Henderson, D. Ranasinghe, V. G. Zakrzewski, J. Gao, N. Rega, G. Zheng, W. Liang, M. Hada, M. Ehara, K. Toyota, R. Fukuda, J. Hasegawa, M. Ishida, T. Nakajima, Y. Honda, O. Kitao, H. Nakai, T. Vreven,

K. Throssell, J. A. Montgomery, Jr., J. E. Peralta, F. Ogliaro, M. J. Bearpark, J. J. Heyd, E. N. Brothers, K. N. Kudin, V. N. Staroverov, T. A. Keith, R. Kobayashi, J. Normand, K. Raghavachari, A. P. Rendell, J. C. Burant, S. S. Iyengar, J. Tomasi, M. Cossi, J. M. Millam, M. Klene, C. Adamo, R. Cammi, J. W. Ochterski, R. L. Martin, K. Morokuma, O. Farkas, J. B. Foresman, and D. J. Fox, Gaussian, Inc., *Wallingford CT*, **2016**.

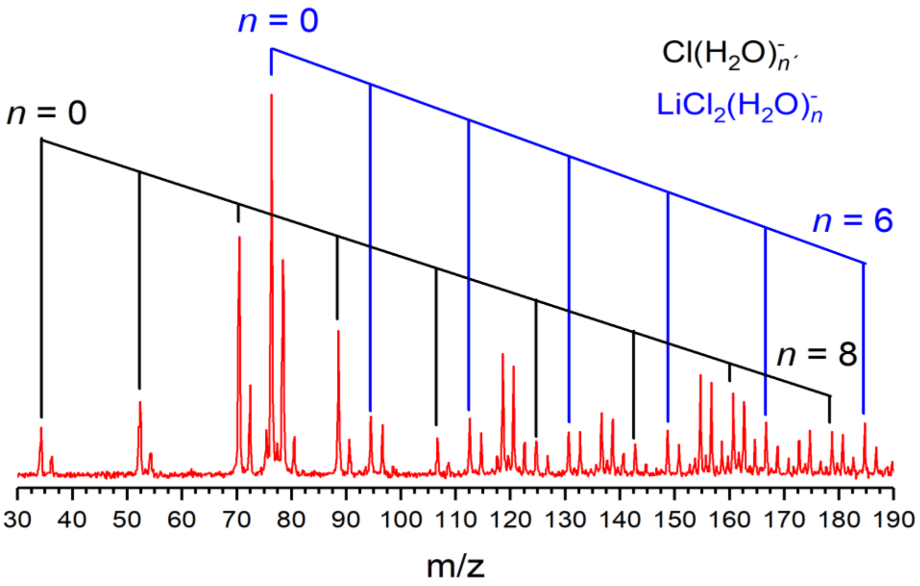

Figure S1. Quadrupole mass spectrum of microhydrated lithium dichloride $[LiCl_2(H_2O)_n]^-$ and chloride anions $[Cl(H_2O)_{n'}]^-$ in the *m/z* range up to 190 amu obtained by ion spray of a 10 mM lithium chloride (LiCl) in 1:1 water/acetonitrile solution.

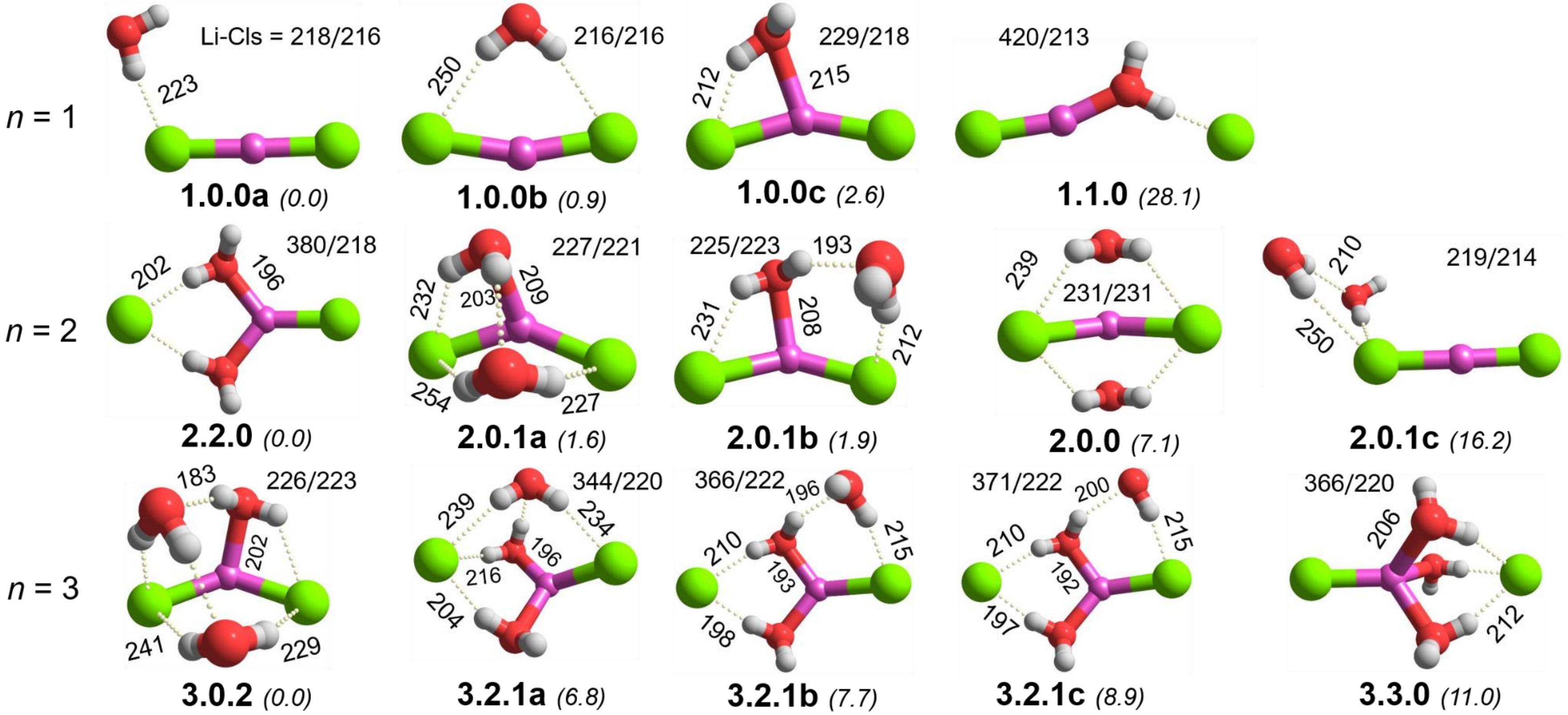

Figure S2. MP2/6-311++G(2df,2pd) structures of $[LiCl_2(H_2O)_n]^-$ (n = 1-3) along with ZPE corrected relative energies ($\Delta E_{0,anh}$, kJ mol^{-1}) and relevant bond distances (in pm).

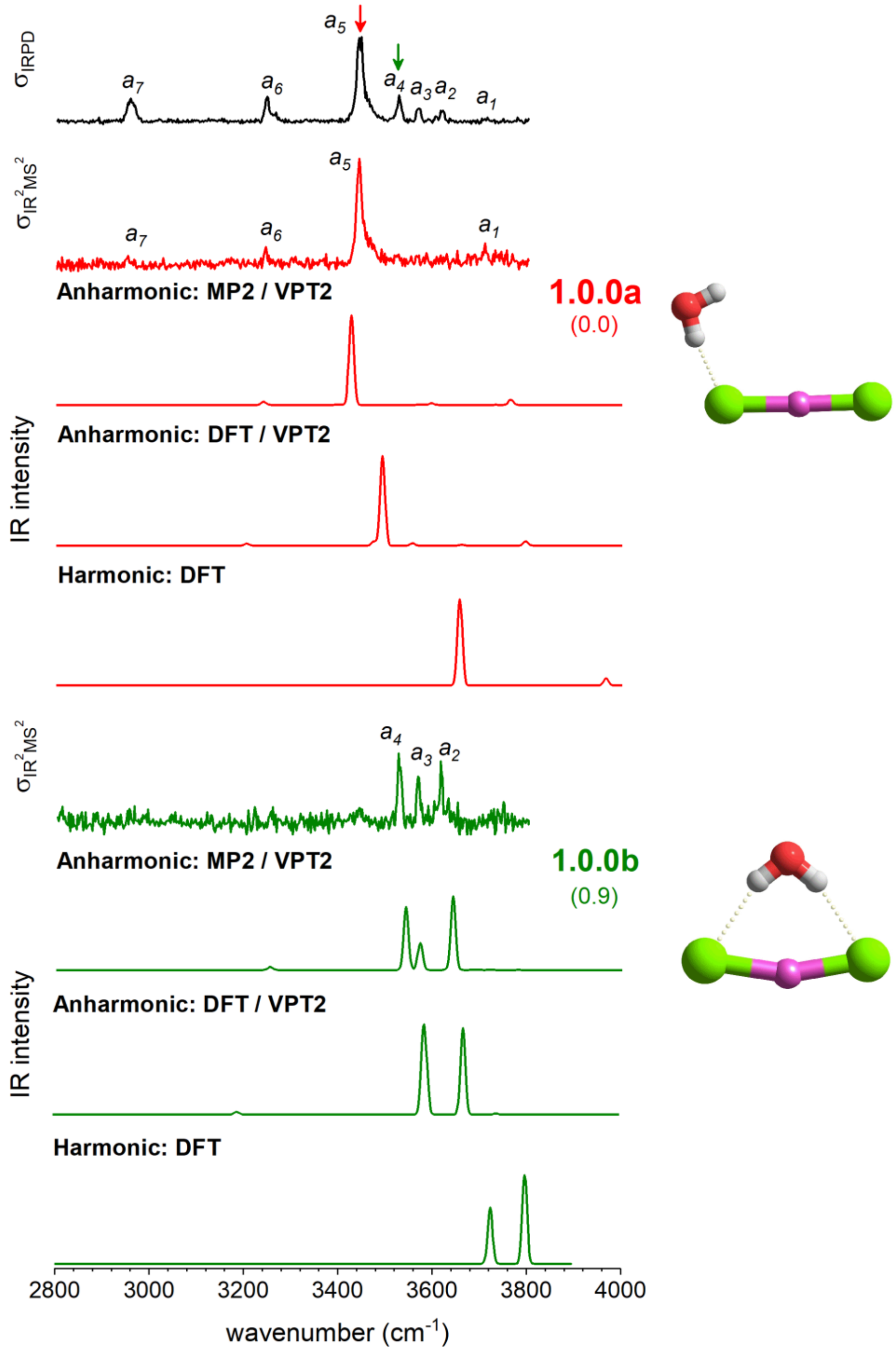

Figure S3. . Comparison of the experimental IRPD and IR^2MS^2 spectra of D_2-tagged $[LiCl_2(H_2O)]^-$ to simulated harmonic (DFT: LC-ωPBE/6-311++G(2df,2pd), scaled by 0.9897) and anharmonic (DFT: VPT2/LC-ωPBE/6-311++G(2df,2pd), MP2: VPT2/MP2/6-311++G(2df,2pd)) spectra of the isomer **1.0.0a** and **1.0.0b**. The probed energies for IR^2MS^2 measurements are designated by color coded arrows in the top trace. Harmonic scaling factor from NIST.

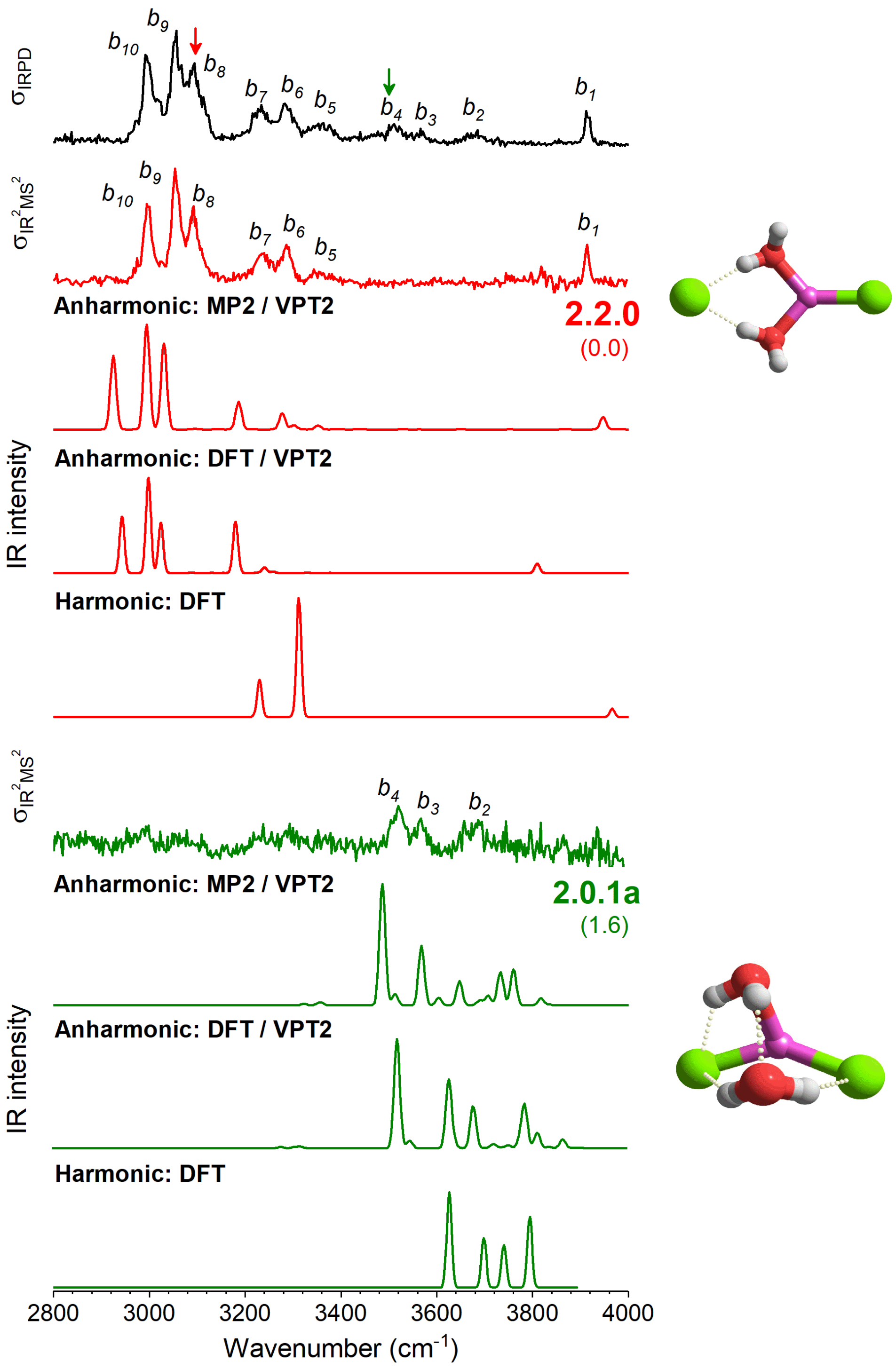

Figure S4. Comparison of the experimental IRPD and IR^{2}MS^2 spectra of D_2-tagged $[LiCl_2(H_2O)_2]^-$ to simulated harmonic (DFT: LC-ωPBE/6-311++G(2df,2pd), scaled by 0.9897) and anharmonic (DFT: VPT2/LC-ωPBE/6-311++G(2df,2pd), MP2: VPT2/MP2/6-311++G(2df,2pd)) spectra of the isomer **2.2.0** and **2.0.1a.** The probed energies for IR^{2}MS^2 measurements are designated by color coded arrows in top trace. Harmonic scaling factor from NIST.

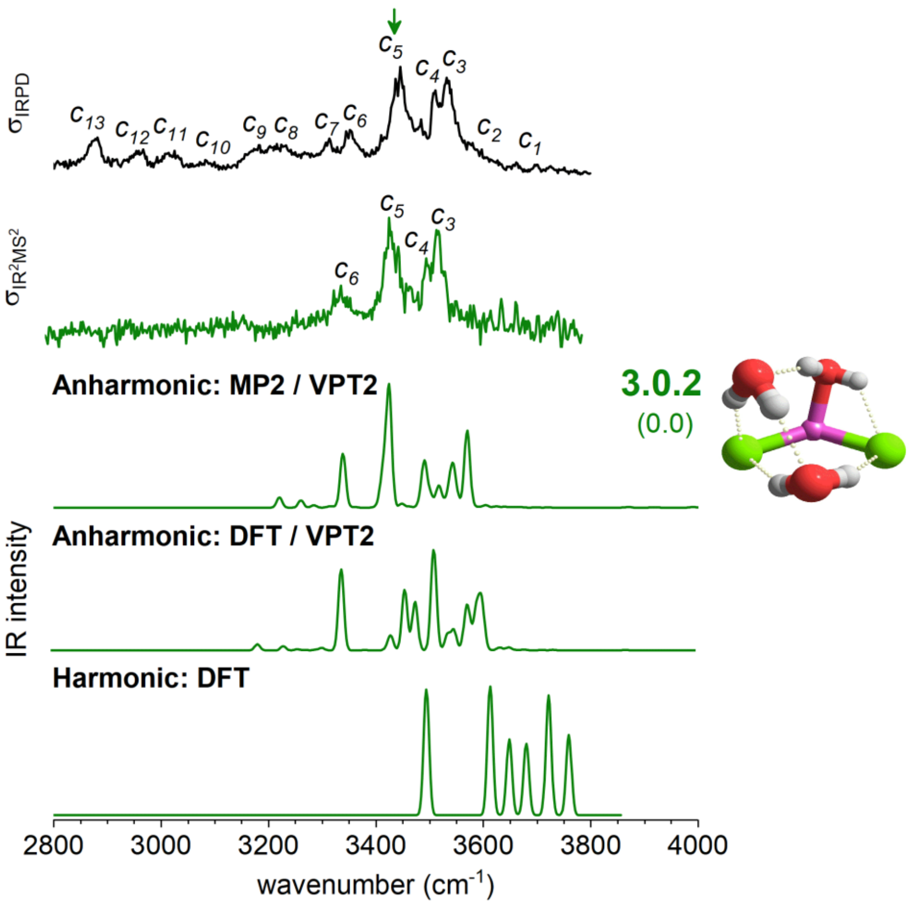

Figure S5. Comparison of the experimental IRPD and IR^{2}MS^2 spectra of D_2-tagged $[LiCl_2(H_2O)_3]^-$ to simulated harmonic (DFT: LC-ωPBE/6-311++G(2df,2pd), scaled by 0.9897) and anharmonic (DFT: VPT2/LC-ωPBE/6-311++G(2df,2pd), MP2: VPT2/MP2/6-311++G(2df,2pd)) spectra of the isomer **3.0.2** The probed energy for IR^{2}MS^2 measurement is designated by color coded arrows in top trace. Harmonic scaling factor from NIST.

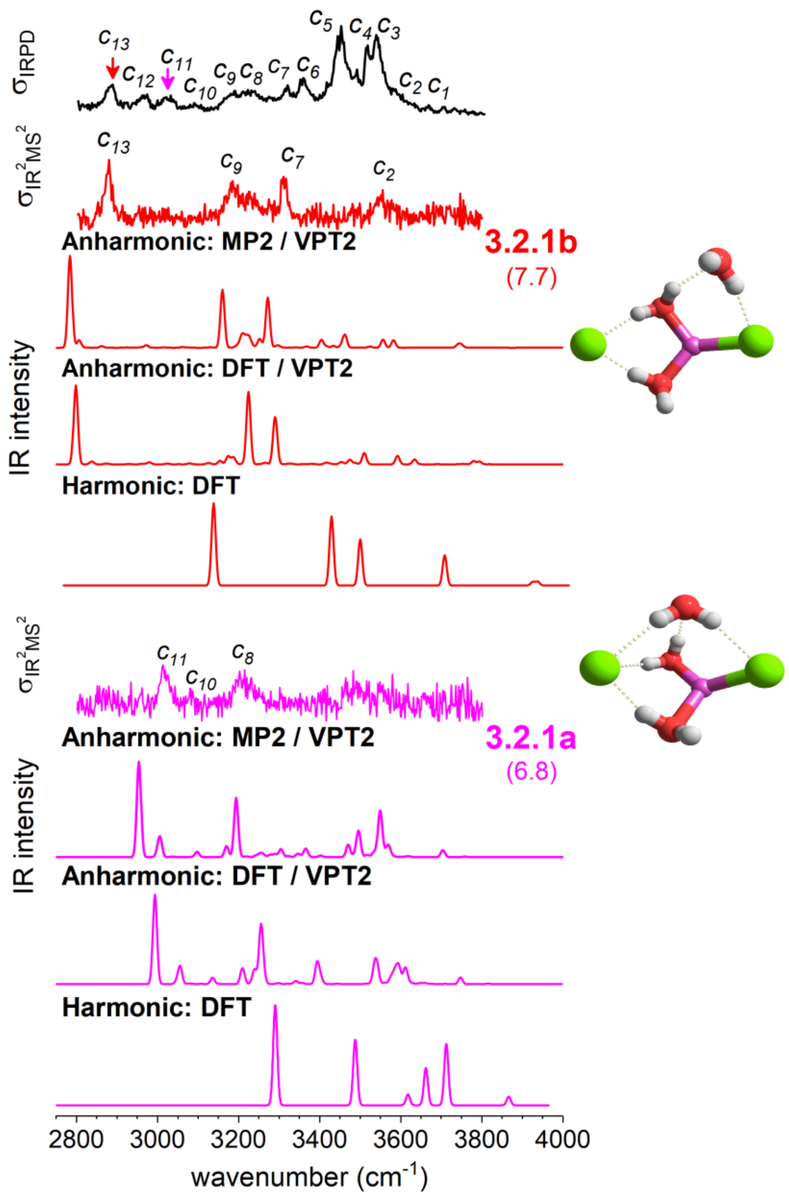

Figure S6. Comparison of the experimental IRPD and IR^2MS^2 spectra of D_2-tagged $[LiCl_2(H_2O)_3]^-$ to simulated harmonic (DFT: LC-ωPBE/6-311++G(2df,2pd), scaled by 0.9897) and anharmonic (DFT: VPT2/LC-ωPBE/6-311++G(2df,2pd), MP2: VPT2/MP2/6-311++G(2df,2pd)) spectra of the isomer **3.2.1a** and **3.2.1b** The probed energy for IR^2MS^2 measurement is designated by color coded arrows in top trace.

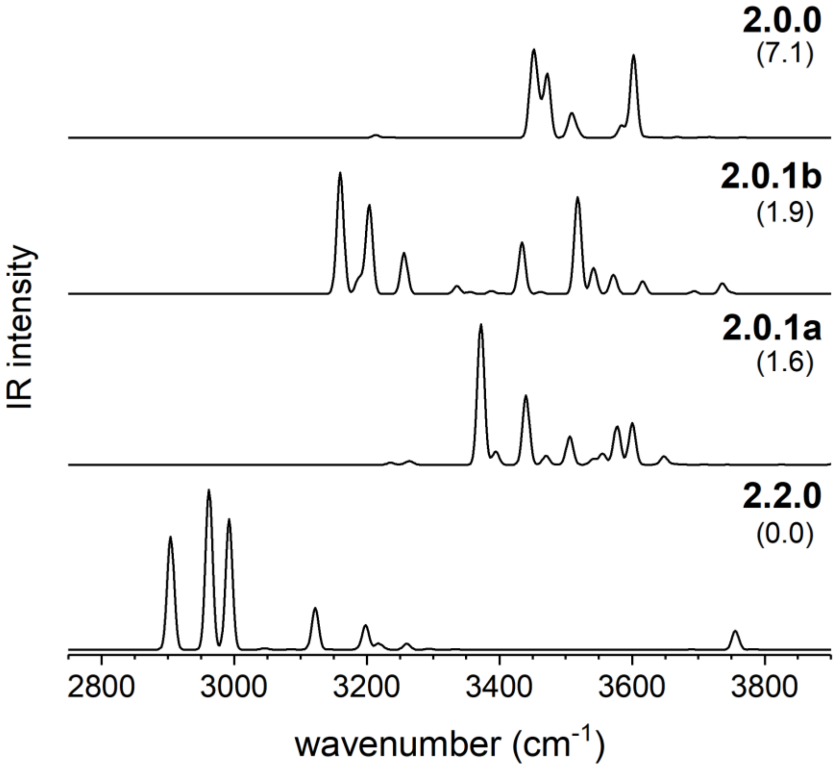

Figure S7. Comparison of the VPT2 / MP2 simulated anharmonic spectra of the lowest energy isomers of $[LiCl_2(H_2O)_2]^-$.

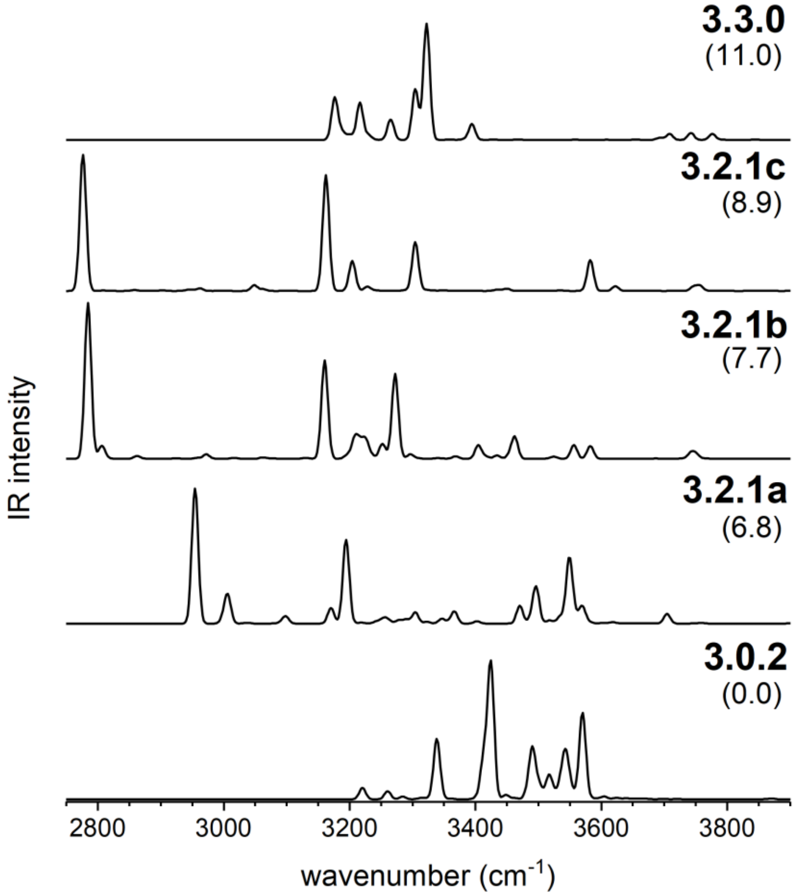

Figure S8. Comparison of the VPT2/MP2 simulated anharmonic spectra of the lowest energy isomers of $[LiCl_2(H_2O)_3]^-$.

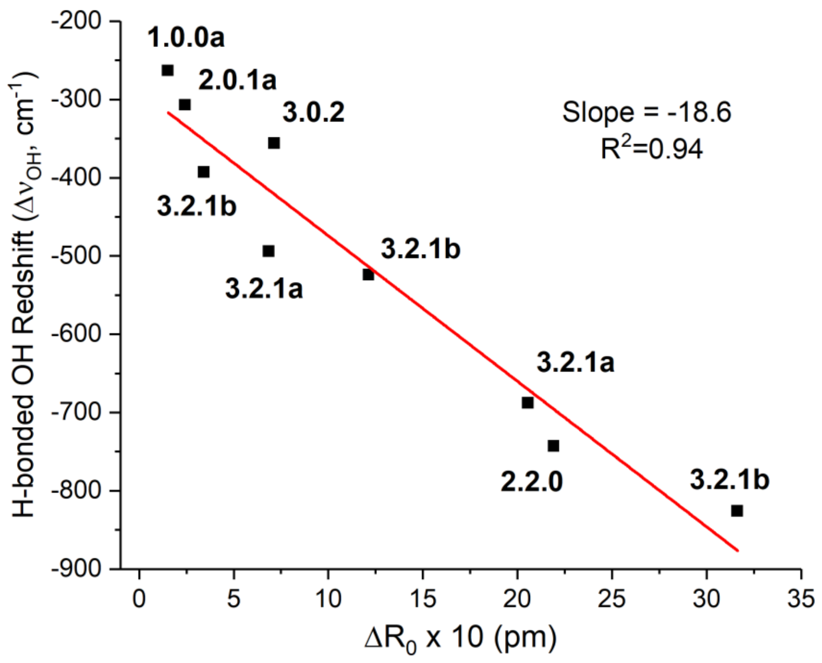

Figure S9. Experimentally obtained redshifts (relative to the averaged OH stretching in a free water molecule) of the hydrogen bonded OH stretches ($\Delta\nu_{OH}$ in cm^{-1}) is plotted as a function of theoretically obtained corresponding increment in vibrationally averaged H-bonded OH bond lengths (ΔR_0 in pm) relative to the OH bond length in a discrete water molecule.

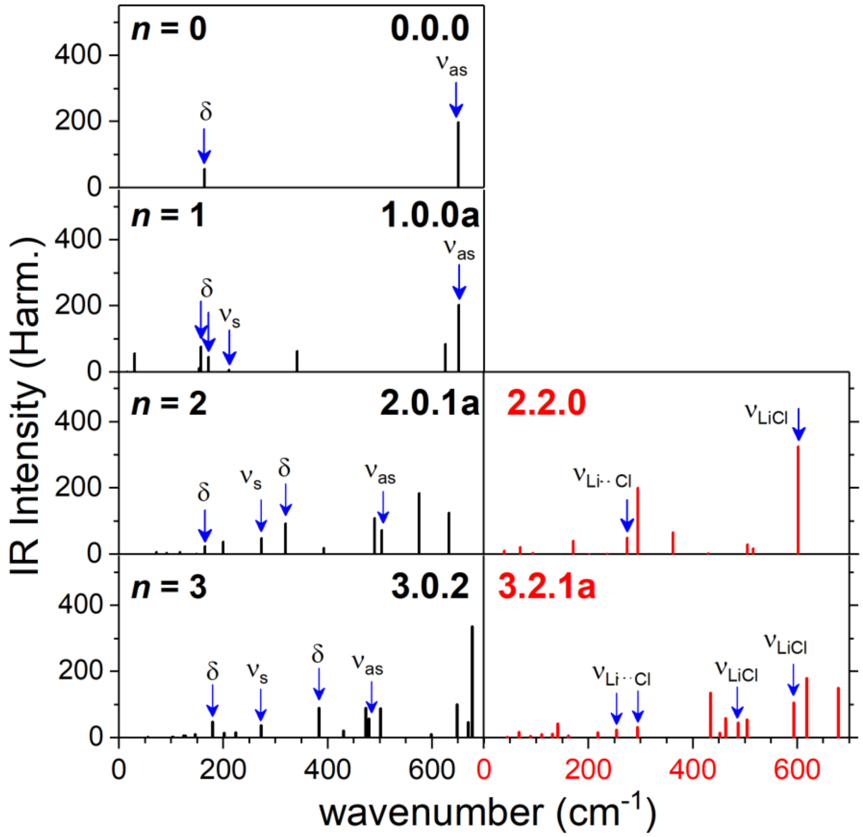

Figure S10. MP2/6-311++G(2df,2pd) harmonic spectra of low energy experimentally identified isomers of $[LiCl_2(H_2O)n]^-$ (n = 1-3) anions in the far-IR low-frequency spectral region derived with MP2/6-311++G(2df,2pd). Intact and solvent-shared structures are plotted in black and red, respectively. Peaks corresponding to interion vibration of intact or solvent-shared $LiCl_2$ core are denoted by arrows (see also table 2).

Table S1. Experimental band positions (in cm^{-1}), anharmonic vibrational frequencies (in cm^{-1}) and band assignments of the features observed in the single-color (IRPD) and two-color (IR^{2}MS^2, probe frequency given in cm^{-1}) vibrational spectra of the D_2-tagged $[LiCl_2(H_2O)_n]^-$ with n = 1-3.

**Label**	**IPRD**	**IR^{2}MS^2**			**Anharm. Frequency^a**			**Assignment^c**
***n* = 1**		**3444^b**	**3529^b**		**1.0.0a**	**1.0.0b**		
a_1	3709	3710			3764			ν_{free}(D-H_2O)
a_2	3616		3618			3642		ν_{as}(DD-H_2O)
a_3	3567		3567			3572		combination
a_4	3529		3526			3542		ν_s(DD-H_2O)
a_5	3444	3442			3426			ν_{HB}(D-H_2O)
a_6	3246	3244			3240			bend overtone
a_7	2958							D_2 stretch
***n* = 2**		**3045^b**	**3394^b**		**2.2.0**	**2.1.0a**		
b_1	3729	3728			3728			ν_{free}(D-H_2O)
b_2	3535		3536			3506		ν_{as}(DD-H_2O)
b_3	3440		3437			3440		ν_s(DD-H_2O)
b_4	3394		3397			3372		ν_s(ADD-H_2O)
b_5	3265	3261			3260			bend overtone
b_6	3203	3205			3198			combination
b_7	3157	3160			3122			combination
b_8	3045	3044			2992			combination
b_9	3014	3011			2962			$\nu_{s,HB}$(D-H_2O's)
b_{10}	2960	2962			2904			$\nu_{as,HB}$(D-H_2O's)
***n* = 3**		**3443^b**	**3016^b**	**2878^b**	**3.0.2**	**3.2.1a**	**3.2.1b**	
c_1	3697<br>3661					3704	3750	ν_{free}(D-H_2O)
c_2	3576			3566			3555	ν_{as} (DD-H_2O)
c_3	3531	3530			3570			ν_{as}(DD-H_2O)
c_4	3508	3509			3541			ν_{as}(ADD-H_2O)
c_5	3443	3442			3423			ν_s(ADD-H_2O)
c_6	3351	3352			3339			ν_s(DD-H_2O)
c_7	3312			3312			3271	ν_s(AD-H_2O)
c_8	3212		3214			3194		ν_s(DD-H_2O)
c_9	3180			3180			3160	ν_s(DD-H_2O)
c_{10}	3079		3076			3006		combination
c_{11}	3016		3018			2953		ν_s-D-H_2O
c_{12}	2964							D_2 stretch
c_{13}	2878			2877			2784	ν_s-D-H_2O

^{a} Anharmonic vibrational frequencies were obtained using the VPT2/MP2/6-311++G(2df,2pd) method for indicated structures (without D_2 tag).

^{b} Probe frequency (in cm^{-1}) at which the IR^{2}MS^2 spectrum was obtained.

[c] OH oscillators are categorized into free (ν_{free}) or hydrogen-bonded (ν_{HBed}) uncoupled OH stretches as well as coupled symmetric (ν_s) or antisymmetric OH stretches (ν_{as}).

Table S2. DFT and MP2 computed relative energies ΔE and ZPE-corrected relative energies ΔE_0 (both in kJ/mol) of low energy isomers for $[LiCl_2(H_2O)_n]^-$ with n = 0 - 3.[a-d]

**Species**	**Label**	**DFT**[a]		**MP2**[c]	
n =		**ΔE**	**ΔE_0**[b]	**ΔE**	**Δ$E_{0,anh}$**[d]
0	0.0.0				
1	1.0.0a	1.2	**0.0**	2.3	**0.0**
	1.0.0b	**0.0**	1.4	**0.0**	0.9
	1.0.0c	2.3	3.9	1.6	2.6
	1.1.0	27.4	25.9	32.6	28.1
2	2.2.0	**0.0**	**0.0**	1.3	**0.0**
	2.0.1a	0.5	3.7	**0.0**	1.6
	2.0.1b	2.3	4.1	1.5	1.9
	2.0.0	6.4	8.4	6.3	7.1
	2.0.1c	17.8	15.1	20.5	16.2
3	3.0.2	**0.0**	**0.0**	**0.0**	**0.0**
	3.2.1a	7.2	6.3	7.9	6.8
	3.2.1b	8.8	4.8	11.8	7.7
	3.2.1c	10.7	5.8	14.0	8.9
	3.3.0	17.7	12.5	16.6	11.0

a LC-ωPBE / 6-311++G(2df,2pd)

b ZPE-corrected energies derived from harmonic DFT frequencies.

c MP2/6-311++G(2df,2pd)

d ZPE-corrected energies derived from anharmonic MP2/VPT2 frequencies.

Table S3. MP2/6-311++G(2df,2pd) harmonic frequencies (cm^{-1}) and intensities (in mol km^{-1}, in parentheses) of interion "stretching" (ν) and "bending" (δ) vibrational modes. (ν_{as}: Cl-Li-Cl antisymmetric stretch, ν_s: Cl-Li-Cl symmetric stretch, δ: Cl-Li-Cl bend, ν_{LiCl}, $\nu_{Li\cdots Cl}$: Li-Cl stretch).

isomer	ν_{as}	ν_s	δ
**0.0.0**	650 (199)	208 (0)	165 (57), 165 (57)
**1.0.0a**	651 (209)	211 (7)	172 (47), 157 (77)
**1.0.0b**	657 (158)	255 (32)	154 (47), 103 (25)
**2.0.1a**	504 (72)	273 (49)	320 (93)*, 165 (24)
**3.0.2**	479 (57)	273 (36)	383 (90)*, 179 (48)
	ν_{LiCl}	$\nu_{Li\cdots Cl}$	
**2.2.0**	601 (325)	275 (50)	
**3.2.1a**	593 (106)*, 487 (45)*	254 (24)	
**3.2.1b**	561 (103)*, 522 (29)*	252 (21)	

* Coupled to $Li^+(\cdot\cdot OH_2)_n$ framework.

Table S4: Value for the cosine similarity (S) between the IR^2MS^2 spectra and calculated VPT2/MP2 anharmonic spectra for $[LiCl_2(H_2O)_n]^-$ (n = 1-3) isomers.[a]

$[LiCl_2(H_2O)_n]^-$	λ_{probe}	Structures	S
n = 1	3444 cm^{-1}	**1.0.0a**	**0.80**
		**1.0.0b**	0.20
		**1.0.0c**	0.14
	3529 cm^{-1}	**1.0.0a**	0.17
		**1.0.0b**	**0.60**
		**1.0.0c**	0.13
2	3045 cm^{-1}	**2.2.0**	**0.88**
		**2.0.1a**	0.08
		**2.0.1b**	0.22
	3394 cm^{-1}	**2.2.0**	0.36
		**2.0.1a**	**0.54**
		**2.0.1b**	0.46
3	3543 cm^{-1}	**3.0.2**	**0.79**
		**3.2.1a**	0.41
		**3.2.1b**	0.28
		**3.2.1c**	0.24
		**3.3.0**	0.34
	3016 cm^{-1}	**3.0.2**	0.23
		**3.2.1a**	**0.52**
		**3.2.1b**	0.30
		**3.2.1c**	0.31
		**3.3.0**	0.38
	2878 cm^{-1}	**3.0.2**	0.38
		**3.2.1a**	0.39
		**3.2.1b**	**0.56**
		**3.2.1c**	**0.56**
		**3.3.0**	0.24

[a]Cosine similarity[7] (S) has been employed to evaluate the agreement between anharmonic calculated and measured IR^2MS^2 spectra. This is an assessment for the similarity of two vectors, as follows: $S = \frac{\sum a_i \cdot c_i}{\sqrt{\sum a_i^2} \cdot \sqrt{\sum c_i^2}}$

The intensities of the IR^2MS^2 spectrum are interpreted as vector a_i and the intensities of the calculated spectrum are interpreted as vector c_i. S-value towards 1 conveys better agreement while

value towards 0 indicate poor agreement. The values for S given refer only to the region (2750 to 3750 cm^{-1}).

Table S5: Cartesian coordinates (in Ångstrom) for the MP2/6-311++G(2df,2pd) optimized structure of $[LiCl_2(H_2O)_n]^-$ where n = 1-3.

n = 1

1.0.0a

	X	Y	Z
Li	0.866375000000	-0.380629000000	0.000216000000
Cl	-1.186398000000	-1.112384000000	-0.000045000000
O	-3.240640000000	1.330957000000	0.000115000000
H	-2.695807000000	0.523152000000	0.000246000000
H	-2.576799000000	2.021676000000	-0.000952000000
Cl	2.868668000000	0.403526000000	-0.000006000000

1.0.0b

Li	0.000918000000	-0.871667000000	0.002658000000
Cl	2.128062000000	-0.524296000000	-0.000228000000
Cl	-2.126649000000	-0.526191000000	-0.000223000000
O	-0.002936000000	2.166606000000	0.000710000000
H	-0.762754000000	1.568526000000	-0.003102000000
H	0.759451000000	1.571895000000	-0.002871000000

1.0.0c

Li	0.196331000000	-0.090057000000	-0.222285000000
Cl	2.351988000000	-0.328698000000	0.016290000000
Cl	-1.987937000000	-0.747514000000	0.019729000000
O	-0.629827000000	1.890227000000	-0.097043000000
H	-1.389228000000	1.286553000000	0.049860000000
H	-0.350001000000	2.157402000000	0.781032000000

1.1.0

Li	1.117938000000	0.111460000000	-0.020401000000
Cl	-3.051992000000	-0.363985000000	0.003741000000
O	-0.511577000000	0.954797000000	-0.021045000000
H	-0.739833000000	1.873393000000	0.116715000000
H	-1.416900000000	0.469181000000	-0.022432000000
Cl	3.222318000000	-0.242799000000	0.004216000000

n = 2

2.2.0

Li	0.958663000000	0.000005000000	0.354263000000
O	-0.299484000000	1.493174000000	0.454428000000
H	-0.138033000000	2.191554000000	-0.181772000000
H	-1.193549000000	1.130275000000	0.219776000000
O	-0.299479000000	-1.493172000000	0.454430000000
H	-1.193532000000	-1.130292000000	0.219699000000
H	-0.137978000000	-2.191570000000	-0.181738000000
Cl	3.058765000000	0.000002000000	-0.219525000000
Cl	-2.789423000000	-0.000002000000	-0.275158000000

2.0.1a

Cl	-2.203407000000	-0.614562000000	-0.153972000000
Cl	1.967741000000	-0.874499000000	0.170011000000
H	0.263413000000	1.794544000000	-0.495874000000
H	1.464932000000	1.049405000000	-1.014413000000
H	-1.021279000000	0.799408000000	1.175808000000
H	0.464400000000	0.691887000000	1.489135000000
Li	-0.076729000000	-0.435278000000	-0.725355000000
O	0.631417000000	1.442394000000	-1.322850000000
O	-0.248286000000	1.343184000000	1.416443000000

2.0.1b

Li	0.308426000000	-0.575778000000	0.471016000000
Cl	-1.806796000000	-1.221619000000	0.134470000000
Cl	2.400813000000	-0.444484000000	-0.348027000000
O	0.454920000000	1.368233000000	1.172917000000
H	1.294298000000	1.292311000000	0.689175000000
H	-0.178728000000	1.734063000000	0.533591000000
O	-1.593148000000	1.713828000000	-0.781133000000
H	-1.837558000000	0.804228000000	-0.497638000000
H	-1.195767000000	1.563986000000	-1.641975000000

2.0.0

Li	-0.000026000000	-0.000120000000	0.605917000000
Cl	-2.085365000000	0.000073000000	-0.390973000000
Cl	2.085203000000	-0.000064000000	-0.391048000000
O	0.000298000000	2.095316000000	0.687676000000
H	0.769622000000	1.933423000000	0.118526000000
H	-0.769249000000	1.934569000000	0.118584000000
O	0.000020000000	-2.095470000000	0.687587000000
H	0.769435000000	-1.933160000000	0.118630000000
H	-0.769516000000	-1.933398000000	0.118756000000

2.0.1c

Li	-1.525821000000	-0.318034000000	0.203816000000
Cl	-3.521695000000	0.205790000000	-0.364651000000
Cl	0.541503000000	-0.746783000000	0.798720000000
O	2.429873000000	1.720689000000	0.298908000000
H	1.824341000000	1.009976000000	0.584732000000

H	1.871639000000	2.262508000000	-0.261582000000
O	3.290350000000	-0.744271000000	-1.146056000000
H	3.293749000000	0.157742000000	-0.803293000000
H	2.489218000000	-1.090589000000	-0.733288000000

n = 3

3.0.2

Cl	-1.725824000000	-1.114666000000	-0.764348000000
Cl	2.334841000000	-0.452350000000	-0.083749000000
H	-0.230406000000	1.954359000000	-0.418438000000
H	1.263589000000	1.666175000000	-0.665634000000
H	-1.928244000000	0.948957000000	0.307600000000
H	-1.003180000000	1.093142000000	1.469009000000
H	-0.647380000000	-1.009327000000	1.385223000000
H	0.778784000000	-0.551594000000	1.588689000000
Li	0.305152000000	-0.227770000000	-1.066704000000
O	0.422648000000	1.790317000000	-1.128568000000
O	-1.517369000000	1.642681000000	0.851272000000
O	-0.093018000000	-0.530389000000	2.021208000000

3.2.1a

Li	0.751344000000	0.661063000000	0.897245000000
O	-0.244720000000	2.131173000000	0.073340000000
H	0.273216000000	2.194031000000	-0.735096000000
H	-1.040519000000	1.603449000000	-0.191024000000
O	-0.406516000000	-0.637201000000	1.789443000000
H	-1.233092000000	-0.414213000000	1.309415000000
H	-0.115422000000	-1.408396000000	1.270471000000
Cl	2.534439000000	0.158261000000	-0.292902000000
Cl	-2.340020000000	0.059499000000	-0.485296000000
O	0.174489000000	-2.073418000000	-0.561314000000

H	-0.579549000000	-1.517825000000	-0.819684000000
H	0.950190000000	-1.506595000000	-0.708188000000

3.2.1b

Li	-0.486916000000	-0.791592000000	0.595774000000
O	0.992101000000	-1.936133000000	0.092023000000
H	0.879198000000	-2.371951000000	-0.754503000000
H	1.722654000000	-1.273041000000	-0.065809000000
O	0.292751000000	0.869764000000	1.196698000000
H	1.176066000000	0.884617000000	0.762619000000
H	-0.242084000000	1.539412000000	0.741890000000
Cl	-2.579096000000	-0.823849000000	-0.149002000000
Cl	2.873588000000	0.314672000000	-0.339232000000
O	-1.729609000000	2.165285000000	-0.364494000000
H	-2.147712000000	1.279837000000	-0.309126000000
H	-1.375669000000	2.180590000000	-1.256243000000

3.2.1c

Li	0.751344000000	0.661063000000	0.897245000000
O	-0.244720000000	2.131173000000	0.073340000000
H	0.273216000000	2.194031000000	-0.735096000000
H	-1.040519000000	1.603449000000	-0.191024000000
O	-0.406516000000	-0.637201000000	1.789443000000
H	-1.233092000000	-0.414213000000	1.309415000000
H	-0.115422000000	-1.408396000000	1.270471000000
Cl	2.534439000000	0.158261000000	-0.292902000000
Cl	-2.340020000000	0.059499000000	-0.485296000000
O	0.174489000000	-2.073418000000	-0.561314000000
H	-0.579549000000	-1.517825000000	-0.819684000000
H	0.950190000000	-1.506595000000	-0.708188000000

3.3.0

Li	0.908418000000	0.001214000000	0.000696000000
O	-0.216157000000	-1.173404000000	-1.276274000000
H	-0.092034000000	-0.856915000000	-2.173152000000
H	-1.125459000000	-0.894473000000	-1.033812000000
O	-0.217528000000	-0.520140000000	1.655230000000
H	-0.093892000000	-1.456085000000	1.824696000000
H	-1.126292000000	-0.447849000000	1.291450000000
O	-0.217603000000	1.693664000000	-0.376515000000
H	-1.126490000000	1.342437000000	-0.258091000000
H	-0.095674000000	2.310676000000	0.347637000000
Cl	3.113261000000	0.000109000000	-0.000351000000
Cl	-2.751797000000	-0.000249000000	-0.000846000000
